# The TIGIT^+^ T regulatory cells subset associates with nosocomial infection and fatal outcome in COVID-19 patients under mechanical ventilation

**DOI:** 10.1038/s41598-023-39924-7

**Published:** 2023-08-21

**Authors:** Mikhael Haruo Fernandes de Lima, Caio Cavalcante Machado, Daniele Carvalho Nascimento, Camila Meirelles S. Silva, Juliana Escher Toller-Kawahisa, Tamara Silva Rodrigues, Flavio Protassio Veras, Marjorie Cornejo Pontelli, Italo A. Castro, Dario Simões Zamboni, José-Carlos A. Filho, Thiago M. Cunha, Eurico Arruda, Larissa Dias da Cunha, Renê D. R. Oliveira, Fernando Q. Cunha, Paulo Louzada-Junior

**Affiliations:** 1 Divisions of Clinical Immunology, Emergency, Infectious Diseases and Intensive Care Unit, Ribeirão Preto Medical School, University of São Paulo, Av. Bandeirantes 3900, Ribeirão Preto, São Paulo 14049-900 Brazil; 2 Center of Research in Inflammatory Diseases, Ribeirão Preto Medical School, University of São Paulo, Ribeirão Preto, São Paulo Brazil; 3 Departament of Pharmacology, Ribeirão Preto Medical School, University of São Paulo, Av. Bandeirantes 3900, Ribeirão Preto, São Paulo 14049-900 Brazil; 4 Department of Cell and Molecular Biology, Ribeirão Preto Medical School, University of São Paulo, Ribeirão Preto, São Paulo Brazil; 5 Virology Research Center, Ribeirão Preto Medical School, University of São Paulo, Ribeirão Preto, São Paulo Brazil

**Keywords:** T cells, Immunology, Adaptive immunity, Infectious diseases, Inflammation, Lymphocytes

## Abstract

The TIGIT^+^FOXP3^+^Treg subset (TIGIT^+^Tregs) exerts robust suppressive activity on cellular immunity and predisposes septic individuals to opportunistic infection. We hypothesized that TIGIT^+^Tregs could play an important role in intensifying the COVID-19 severity and hampering the defense against nosocomial infections during hospitalization. Herein we aimed to verify the association between the levels of the TIGIT^+^Tregs with the mechanical ventilation requirement, fatal outcome, and bacteremia during hospitalization. TIGIT^+^Tregs were immunophenotyped by flow cytometry from the peripheral blood of 72 unvaccinated hospitalized COVID-19 patients at admission from May 29th to August 6th, 2020. The patients were stratified during hospitalization according to their mechanical ventilation requirement and fatal outcome. COVID-19 resulted in a high prevalence of the TIGIT^+^Tregs at admission, which progressively increased in patients with mechanical ventilation needs and fatal outcomes. The prevalence of TIGIT^+^Tregs positively correlated with poor pulmonary function and higher plasma levels of LDH, HMGB1, FGL2, and TNF. The non-survivors presented higher plasma levels of IL-33, HMGB1, FGL2, IL-10, IL-6, and 5.54 times more bacteremia than survivors. Conclusions: The expansion of the TIGIT^+^Tregs in COVID-19 patients was associated with inflammation, lung dysfunction, bacteremia, and fatal outcome.

## Introduction

At the beginning of the COVID-19 pandemic, the major challenge imposed by SARS-CoV-2 infection was the large number of patients needing mechanical ventilation^1,2^. Although the COVID-19 vaccines effectively reduced hospitalization, deaths due to severe SARS-CoV-2 infection remain^3^. It is well-recognized that admission to intensive care units (ICU) increases by up to 37% the risk of developing sepsis, which represents the leading cause of death in ICU. Sepsis is characterized by a life-threatening organ dysfunction caused by a dysregulated immune response triggered by infection^4^. Beyond the well-known risk factors for the severe forms of COVID-19, such as obesity, metabolic disorders, and cardiovascular diseases^5–8^, it has been demonstrated that the development of nosocomial infection and sepsis represents the second most important cause of death after respiratory failure^9,10^. Thus, a better understanding of the immunomodulatory mechanisms behind COVID-19 is essential for treating hospitalized patients, whether in preventing respiratory failure or treating sepsis associated with nosocomial infections.

The T regulatory cells (Tregs) expressing the transcription factor FOXP3 are a vital gatekeeper for immune homeostasis, self-tolerance, and control of inflammation^11^. Although conflicting reports, it has been suggested that perturbation of the Treg repertoire correlates with COVID-19 severity by inhibiting the anti-viral immunity and an exacerbated inflammatory response^2,12–14^. Recognizing that the T cell Ig and ITIM domain receptor (TIGIT)-expressing FOXP3^+^ Tregs (TIGIT^+^Tregs) represent a distinct subpopulation of Tregs that exerts a robust suppressive activity on cellular immunity^15,16^, we have previously demonstrated that increased TIGIT^+^Tregs levels predispose septic individuals to opportunistic infections^17^. Thus, we hypothesized that TIGIT^+^Tregs could play an important role in intensifying COVID-19 severity by hampering the defense mechanisms against nosocomial infections during ICU hospitalization. Herein, we performed immunophenotyping of TIGIT^+^ and TIGIT^-^ Treg subsets in blood samples collected from unvaccinated patients with COVID-19 at their hospital admission from May 29th to August 6th, 2020, in an attempt to identify whether increased levels of TIGIT^+^Tregs could imply mechanical ventilation requirement and increased susceptibility to nosocomial infection during hospitalization.

## Material and methods

All experiments were approved and performed in accordance with relevant guidelines and regulations of the National Ethics Committee, Brazil (#30,248,420.9.0000.5440).

### Patients

Seventy-two adult patients who tested positive for COVID-19 using RT-PCR were enrolled in this study. The blood samples were collected at admission in Hospital das Clínicas, Faculdade de Medicina de Ribeirão Preto da Universidade de São Paulo, from May 29th to August 6th, 2020. The exclusion criteria were: the use of immunosuppressive therapy; metastatic cancer; pregnancy or lactation; and inability to understand the consent form. We obtained blood samples from a healthy control group composed of 28 age and sex-matched subjects. The clinical and laboratory features are listed in Table 1.

**Table 1:** Clinical and laboratory data of COVID-19 patients at admission according to the mechanical ventilation therapy and outcome.

	No mechanical ventilation	Mechanical ventilation	Mechanical ventilation survivors	Mechanical ventilation non-survivors
Demographics				
Number	46	26	13	13
Age—yr ± SD	52.25 ± 15.76	61.76 ± 16.45*	62.83 ± 10.07	67.12 ± 12.41
Female—n° (%)	23 (50)	5 (19)	3 (23)	2 (15)
Respiratory status				
Mechanical ventilation—nº (%)	0	26 (100)	13 (100)	13 (100)
Nasal-cannula oxygen—n° (%)	46 (100)	0 (0)	0 (0)	0 (0)
Room air—n° (%)	0 (0)	0 (0)	0 (0)	0 (0)
PaO_2_/FiO_2_^a^ ± SD	342.5 ± 47.9	249.6 ± 121.9*	255.1 ± 134.8*	244.2 ± 113.4*
Clinical evolution over time—days ± SD				
∆T^b^ symptoms onset and hospitalization	9.47 ± 3.57	11.53 ± 7.31	13.30 ± 7.41	9.76 ± 7.06
∆T^b^ hospital admission and intubation	–	0.91 ± 1.95	1.63 ± 2.46	0.25 ± 1.05
Total hospitalization period	7.8 ± 5.58	22.13 ± 15.65*	21.41 ± 9.40*	22.90 ± 20.98*
Outcome—n° (%)				
Deaths	0	13 (50)	0 (0)	13 (100)
Comorbidities—n° (%)				
Diabetes mellitus	16 (35)	13 (50)	6 (46)	7 (53)
Cardiopathy	5 (11)	6 (23)	4 (31)	2 (15)
Nephropathy	2 (4)	5 (19)	1 (8)	4 (33)
Pneumopathy	8 (17)	2 (8)	–	2 (17)
Autoimmune diseases	–	1 (4)	1 (8)	–
Cancer	1 (2)	2 (8)	2 (15)	–
Stroke	2 (4)	1 (4)	-	1 (8)
Obesity	21 (45)	9 (35)	3 (23)	6 (46)
Arterial hypertension	15 (33)	15 (58)	6 (46)	9 (67)
Immunodeficiency	3 (6)	1 (4)	0	1 (8)
History of smoking	14 (30)	7 (27)	5 (38)	2 (15)
Laboratorial results ± SD				
CRP (mg/dL)^c^	6.35 ± 3.32	10.47 ± 5.27*	7.92 ± 3.92	12.77 ± 5.44*^#^
D-dimer (μg/mL)^d^	1.23 ± 0.61	3.39 ± 2.72*	2.00 ± 1.80	4.56 ± 2.87*^#^
LDH (U/L)^e^	406.63 ± 159.74	836.67 ± 505.54*	584.31 ± 324.02	1089.03 ± 538.92*^#^
Ferritin (ηg/mL)^f^	474.85 ± 540.22	1254.1 ± 706.93*	1356.7 ± 837.82*	1130.98 ± 526.59*
Neutrophils/μL (10^3^) ^g^	6.54 ± 1.98	10.99 ± 6.64*	8.80 ± 3.05	10.88 ± 7.71
Lymphocytes/μL (10^3^)^h^	1.86 ± 0.75	1.45 ± 1.21*	1.20 ± 0.95	1.70 ± 1.42
NLR^i^	3.52 ± 1.26	7.96 ± 6.88*	10.08 ± 8.11	13.25 ± 15.51
Platelets/μL (10^5^)^j^	2.51 ± 0.79	2.09 ± 0.97*	2.50 ± 0.94	1.69 ± 0.86*^#^
Cytokines ± SD				
IL-6 (ρg/mL)	9.17 ± 9.66	26.37 ± 28.11*	17.33 ± 23.75*	35.41 ± 30.27*^#^
IL-10 (ρg/mL)	16.89 ± 16.52	31.48 ± 42.23	11.53 ± 10.99	55.86 ± 53.57*^#^
IL-33 (ρg/mL)	–	–	3.47 ± 1.78	6.61 ± 4.29^#^
FGL2 (ηg/mL)	–	–	1.43 ± 0.68	2.04 ± 0.69^#^
HMGB1 (ρg/mL)	–	–	35.52 ± 23.49	77.7 ± 54.46^#^
IFN-γ (ρg/mL)	11.93 ± 9.91	3.52 ± 4.99*	6.88 ± 8.08	4.45 ± 8.48*
TNF (ρg/mL)	13.95 ± 6.65	12.78 ± 10.28	9.52 ± 4.61	16.04 ± 13.31

^a^PaO_2_/FiO_2_, arterial oxygen tension/fraction of inspired oxygen ratio.

^b^∆T, increment of time between two events.

^c^CRP, C-reactive protein (normal value < 0.5 mg/dl).

^d^D-dimers (normal value < 0.5 μg/ml).

^e^LDH, lactic dehydrogenase (normal range: 120–246 U/liter).

^f^ Ferritin (normal range, 10–291 ng/ml).

^g^Neutrophils normal range 1700–7200/µL.

^h^Lymphocytes normal range 1170–3450/µL.

^i^NLR, neutrophil/lymphocyte ratio.

^j^Platelets normal range 150,000–450,000µL.

*p < 0.05 compared with No Mechanical Ventilation group.

^#^*p* < 0.05 compared with Mechanical Ventilation Survivors group, Mann–Whitney test.

### Plasma and PBMC isolation

Whole blood was collected in tubes containing EDTA (BD Vacutainer CPT). The material was centrifuged at 400×*g* for 10 min at room temperature. The plasma was stored at − 70 °C, and the cell pellet was resuspended in 1X PBS, pH7,4 (Gibco-BRL) for the peripheral mononuclear cells (PBMC) isolation through Ficoll-Paque 1077 (Sigma-Aldrich) following the manufacturer’s protocol. The purified cells were washed, and the pellet was resuspended in X-Vivo 20 media (Lonza) for subsequent flow cytometry analysis.

### Flow cytometry

Aliquots of freshly purified PBMC (1 × 10^6^ cells) were washed and subsequently stained with the surface Treg markers CD3 (FITC, BD #557,694), TIGIT (PE, eBioscience #12-9500-42), CD4 (V450, BD #651,849), and the Fixable Viability Dye eFluor 780 (APCH7, eBioscience #65-0865-14) for 30 min at 4 °C. The cells were subsequently fixed and permeabilized for FOXP3 staining (Alexa 647, BD #561,184) using eBioscience™FOXP3/Transcription Factor Staining Buffer Set (#00-5523-00) following the manufacturer’s protocol. The acquisition of cells was performed in the FACS Verse Cytometer, and the analyses were made using the FlowJo Analysis Software v10.

### Cytokine assays

The levels of 9 cytokines were detected from the plasma of COVID-19 patients using Millipore bead-based flow immunoassays (HCYTA-60 K for IFN-g, IL-4, IL-5, IL-6, IL-10, IL-13, and TNF; HCYP2MAG-62 K for IL-33; HCYP4MAG-64 k for HMGB1) in a Luminex 200 system following the manufacturer’s instructions. The measurement of FGL2 plasma levels was determined using LEGEND MAX™ Human FGL2 ELISA Kit (Biolegend #436,907) and read at 450 nm in a Spectra Max 250 plate reader.

### Statistics

Statistical significance was determined by either a two-tailed paired or unpaired Student t-test for data that reached normal distribution, and the Mann–Whitney test was used for not normally distributed data or one-way ANOVA followed by Tukey’s post hoc test. Spearman’s rank-order correlation (r) was calculated to describe correlations. Statistical analyses and graph plots were performed with GraphPad Prism 8.4.2 software. *P* < 0.05 was considered statistically significant.

### Ethical approval

The study was approved by the National Ethics Committee, Brazil (#30,248,420.9.0000.5440).

### Consent to participate

Informed consent was obtained from all individual participants included in the study.

## Results

### Cohort characteristics

Our cohort was stratified according to the mechanical ventilation requirement during hospitalization and their outcome to evaluate the potential association between our experimental data and COVID-19 patients’ clinical course. It is important to emphasize that all parameters analyzed in this study involved unvaccinated patients during their hospital entrance from May 29th to August 6th, 2020.

The demographic and clinical data of patients are reported in Table 1. The survivor rate was 100% among non-intubated patients and 50% among those that required mechanical ventilation. Although detected a significative difference in age concerning mechanical ventilation requirement, in which intubated patients (n = 26, age 61.76 ± 16.45) were older than non-intubated patients (n = 46, age 52.25 ± 15.76), no significative difference in age was observed among intubated patients that did survive or not (62.83 ± 10.07 and 67.12 ± 12.41, respectively). The female gender was more prevalent among patients that did not require mechanical ventilation during hospitalization. The time of symptoms of COVID-19 before the patients’ admission was similar when stratifying patients as non-intubated vs intubated or survivors vs. non-survivors. As expected, the period of hospitalization among patients that required mechanical ventilation was longer than that of non-intubated patients, and no difference in the days of hospitalization was observed between those intubated patients that survived or passed away. Notably, the mean period taken from admission to intubation was 0.91 ± 1.95 days, which suggests that all experimental findings in this study represent a picture of the immune system moments before mechanical ventilation. Regarding lung dysfunction, we could observe that the mechanical ventilation requirement was associated with lower PaO_2_/FiO_2_ values (249.6 ± 121.9 versus 342.5 ± 47.9) during hospital entrance. No difference in the PaO_2_/FiO_2_ values was observed, stratifying the intubated patients according to their outcomes.

In agreement with already reported data, obesity, systemic arterial hypertension, and diabetes mellitus were the most prevalent comorbidities in all analyzed groups of patients. We detected that diabetes mellitus, cardiopathy, nephropathy, autoimmune diseases, non-metastatic cancer, and systemic arterial hypertension were more prevalent among the patients that required mechanical ventilation compared to non-intubated patients. Stratifying the intubated patients, we could observe that systemic arterial hypertension, obesity, and nephropathy were the most prevalent comorbidities among non-survivors compared with survivors (Table 1). We also confirmed that the patients that required mechanical ventilation presented increased levels of C-reactive protein (CRP), D-dimers, lactate dehydrogenase (LDH), Ferritin, Neutrophil/Lymphocyte cell ratio (NLR), and diminished levels of platelets in peripheral blood. Stratifying the intubated patients according to their survival, we could detect raised levels of CRP, D-dimers, and LDH, as well as diminished amounts of platelets in the blood of the non-survivors during their admission to the hospital (Table 1).

### Diminished levels of IFN-γ accompanied the requirement for mechanical ventilation and increased levels of IL-6 at admission

The severity of COVID-19 is associated with neutrophilia, lymphopenia, an increase in proinflammatory response, and inefficient cellular immunity^1,2^. The requirement for mechanical ventilation during hospitalization was associated with increased neutrophils and reduced lymphocytes count in peripheral blood (Table 1). Regarding patients’ immune response, the need for mechanical ventilation was accompanied by decreased levels of IFN-g and raised levels of IL-6 in the plasma (Table 1, Supplementary Fig. 1A,B). No differences were detected concerning the plasma levels of IL-10 and TNF (Table 1, Supplementary Fig. 1C,D).

### COVID-19 patients exhibit signs of dysregulation of the immune response due to disturbance of the FOXP3^+^ T regulatory cell repertoire

**Figure 1:**
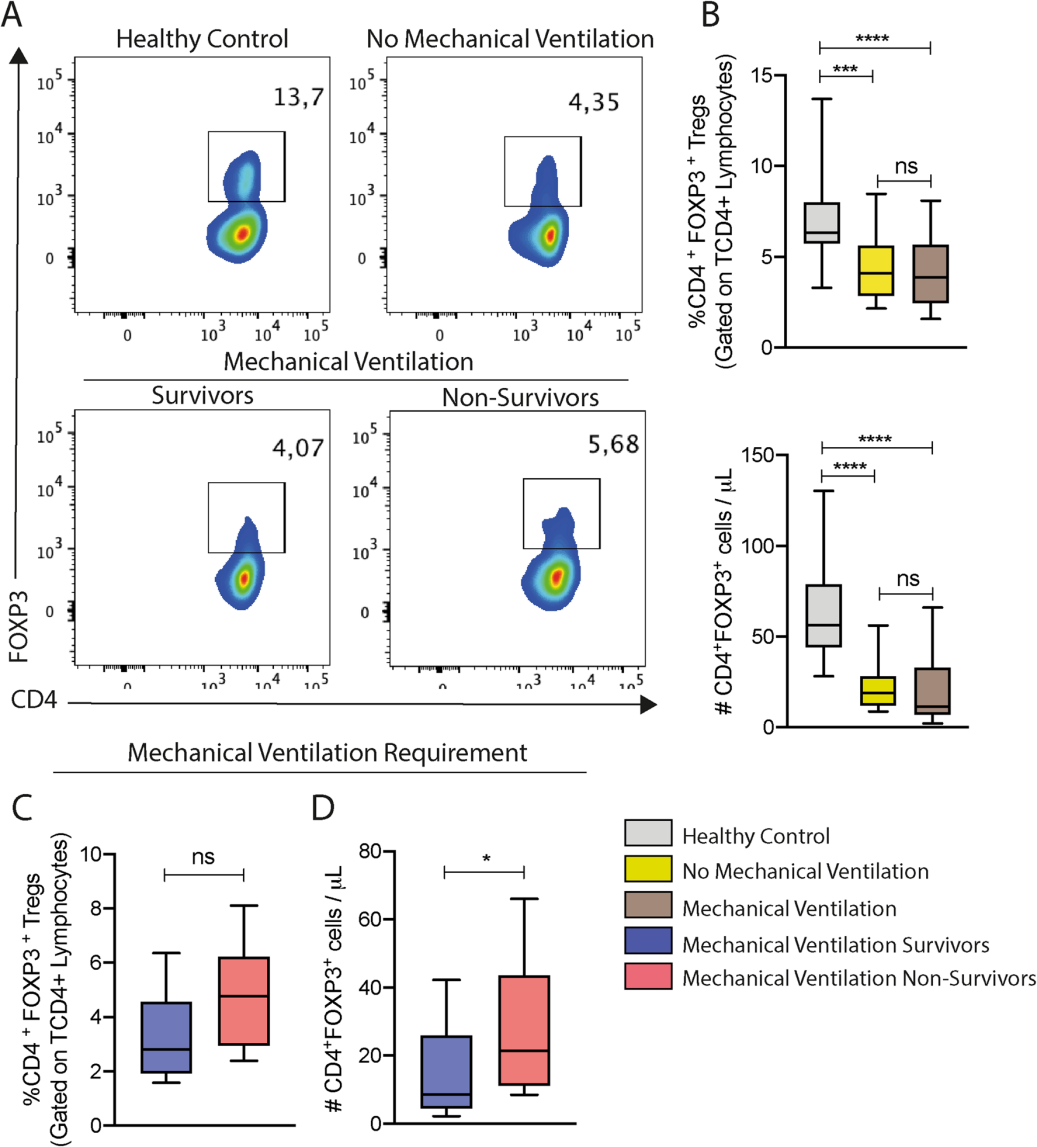
COVID-19 was marked by decreased levels of the FOXP3 + Treg repertoire. (**A**) Blood samples of 42 COVID-19 patients collected at admission and 18 healthy volunteers were evaluated according to the FOXP3^+^Treg repertoire by flow cytometry. The data was expressed in frequency relative to CD4^+^cells (%), and absolute numbers per μL of blood (#). (**B**) Healthy Controls are represented by gray bars (n = 18), patients that did not require mechanical ventilation are represented by the yellow bars (n = 18), and the intubated patients are represented by the brown bars (n = 26). (**C**–**D**) The intubated patients were stratified in survivors (blue bars, n = 13) and non-survivors (red bars, n = 13). Statistical significance was determined by either the one-way ANOVA followed by Tukey’s post hoc test, or the unpaired Student t test for data that reached normal distribution, and the Mann–Whitney test for not normally distributed data. *p < 0.05.

The defective cellular immunity and accentuated proinflammatory response reported in severe COVID-19’patients^3^ prompted us to investigate whether the levels of FOXP3^+^ T regulatory cells at hospital entrance would influence in the requirement of mechanical ventilation during hospitalization and eventually patients’ outcome. Therefore, based on the gating strategy represented in Supplementary Fig. 2A, we observed that both the frequency and absolute number of circulating FOXP3^+^T regulatory cells were reduced in COVID-19 patients compared to healthy controls, and no difference was observed between patients that required or not mechanical ventilation (Fig. 1A,B). Stratifying the intubated patients according to their survival, despite no significant difference in the frequency of FOXP3^+^ T regulatory cell repertoire (Fig. 1A,C), we detected higher circulating cell numbers among the non-survivors (Fig. 1D). The higher FOXP3^+^T regulatory cell numbers observed among the intubated patients who did not survive were independent of the total lymphocyte and the TCD4^+^ cell count in peripheral blood (Supplementary Fig. 2B).

### The TIGIT^+^FOXP3^+^T regulatory cells subset is predominant among intubated COVID-19 patients and associates with fatal outcomes

It has been described that the TIGIT + FOXP3 + T regulatory cells subset exerts strong suppressive properties on cellular immunity^4^ and has an important role during immunosuppression due to sepsis^5^. Thus, we first attempted to quantify the TIGIT^+^ and TIGIT^-^ FOXP3^+^T regulatory cell subsets in the blood of COVID-19 patients based on the gating strategy represented in Supplementary Fig. 2A.

**Figure 2:**
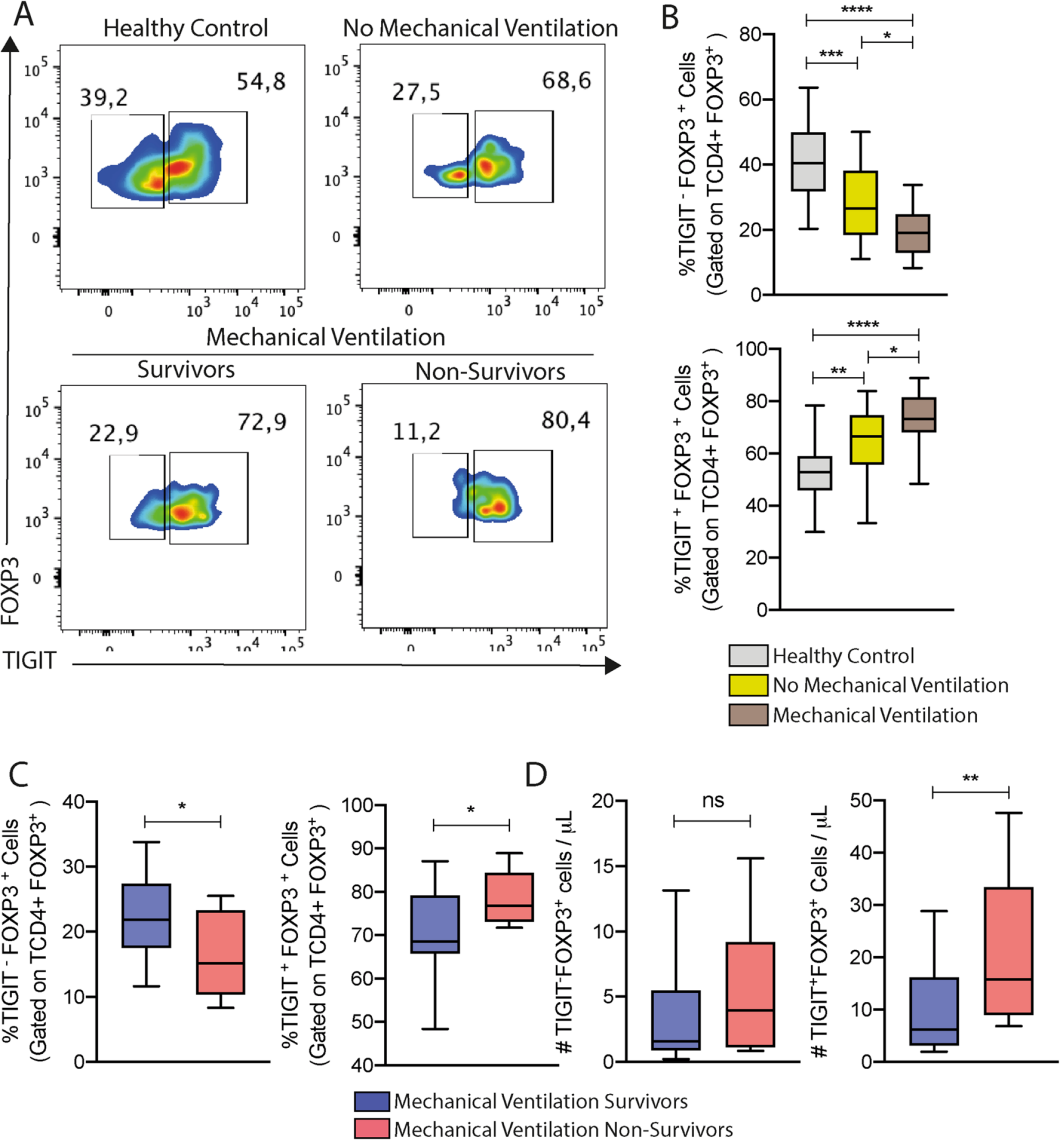
The TIGIT^+^Treg subset was increased in COVID-19 patients with worst prognosis. (**A**) Blood samples of 42 COVID-19 patients collected at admission and 18 healthy volunteers were evaluated according to the TIGIT^+^ and TIGIT^-^ FOXP3^+^Treg subsets by flow cytometry. The data was expressed in frequency relative to FOXP3^+^Treg repertoire (%), and absolute numbers per μL of blood (#). (**B**) Healthy Controls are represented by gray bars (n = 18), patients that did not require mechanical ventilation are represented by the yellow bars (n = 18), and the intubated patients are represented by the brown bars (n = 26). The intubated patients were stratified in survivors (blue bars, n = 13) and non-survivors (red bars, n = 13). (**C**) Frequency of TIGIT^-^FOXP3^+^ Tregs, and Frequency of TIGIT^+^FOXP3^+^Tregs. (**D**) Absolute number of TIGIT^-^FOXP3^+^Tregs and TIGIT^+^FOXP3^+^Tregs subsets. Statistical significance was determined by either the one-way ANOVA followed by Tukey’s post hoc test, or the unpaired Student t test for data that reached normal distribution, and the Mann–Whitney test for not normally distributed data. *p < 0.05.

As shown in Fig. 2A,B, the inflammatory condition due to COVID-19 disturbed the frequencies of the TIGIT^+^ and TIGIT^-^ FOXP3^+^ T regulatory cell subsets. Although the diminished TIGIT^+^ and TIGIT^−^ FOXP3^+^ T regulatory cells count in the blood of COVID-19 patients independently of their respiratory status (Supplementary Fig. 2C), their proportion of both TIGIT^+^ and TIGIT^−^ FOXP3^+^ T regulatory cells subsets were strongly disturbed. Thus, we observed that patients in need of mechanical ventilation presented lesser frequencies of the TIGIT^−^FOXP3^+^Treg cell subset and higher frequencies of the TIGIT^+^FOXP3^+^Treg cell subset than patients that did not require that ventilatory assistance (Fig. 2A,B). Stratifying the intubated patients according to their survival, we observed that the non-survivors presented even lesser frequencies of TIGIT^−^FOXP3^+^T regulatory cells at the expense of expanding the frequencies of TIGIT^+^FOXP3^+^T regulatory cells subset in peripheral blood compared with survivors (Fig. 2C). In terms of absolute cell number, regardless of no significant difference in the TIGIT^−^FOXP3^+^T regulatory, we detected higher numbers of circulating TIGIT^+^FOXP3^+^T regulatory cells at hospital entry for patients that did not survive (Fig. 2D). These findings corroborate previous reports describing the dysregulation of the immune system among COVID-19 patients with the worst outcome and suggest that the immunoregulatory mechanisms performed by FOXP3^+^T regulatory cells are biased, favoring the TIGIT^+^ Treg subset.

### The increase of the TIGIT^+^FOXP3^+^T regulatory cells correlates with lung dysfunction, highly inflammatory response, and cell death

**Figure 3:**
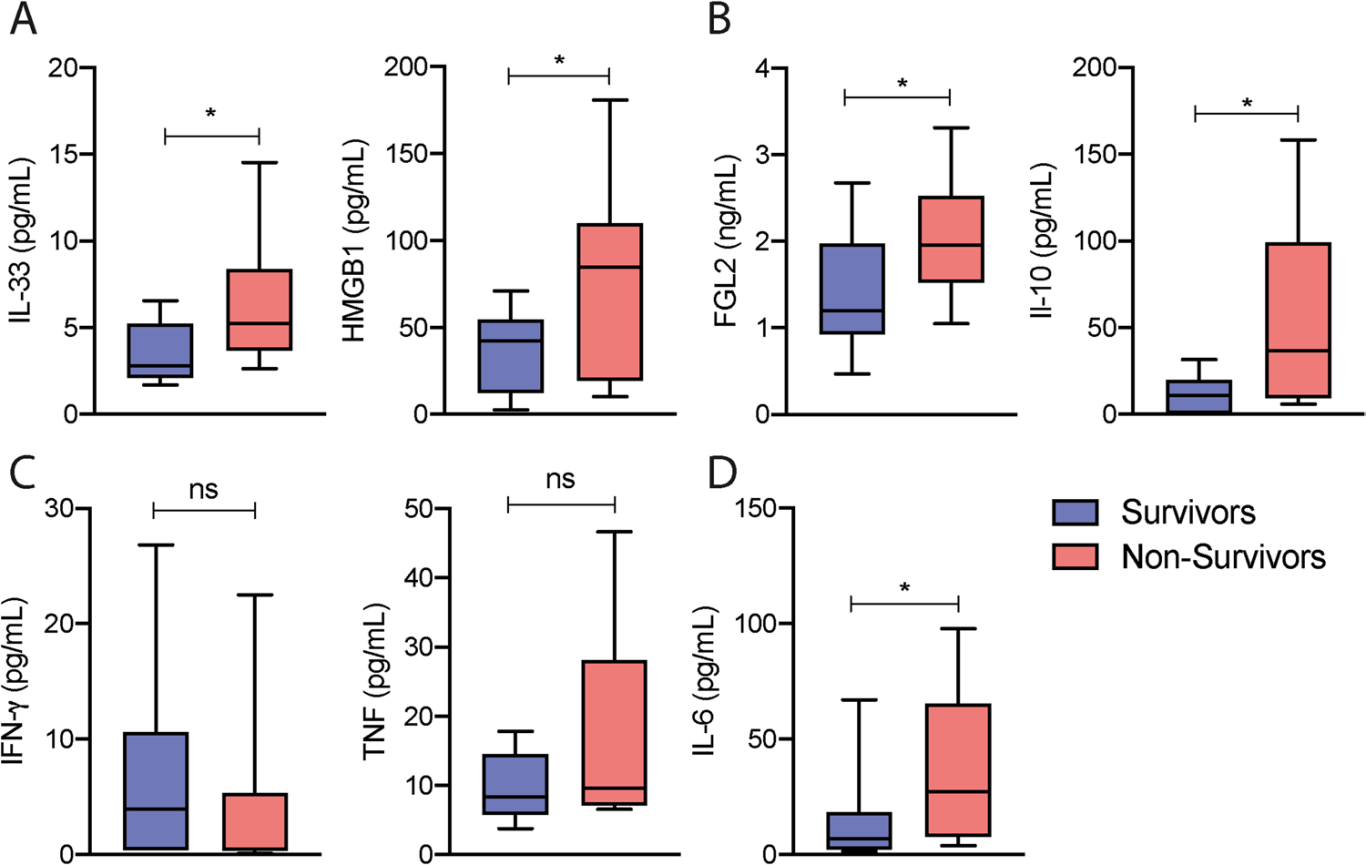
Cytokine measurement of intubated COVID-19 patients stratified in survivors and non-survivors. The cohort of the intubated COVID-19 patients (n = 26) were evaluated according to their cytokine levels in the plasma at admission. The intubated patients were stratified in survivors (blue bars, n = 13) and non-survivors (red bars, n = 13). (**A**) Interleukin-33, IL-33; High Mobility Group Box 1, HMGB1; (**B**) Fibrinogen-like 2, FGL2; Interleukin-10, IL-10; (**C**) Interferon-γ, IFN-γ; Tumor necrosis fator, TNF; (**D**) Interleukin-6, IL-6. Statistical significance was determined by either the unpaired Student t test for data that reached normal distribution, and the Mann–Whitney test for not normally distributed data. *p < 0.05.

**Figure 4:**
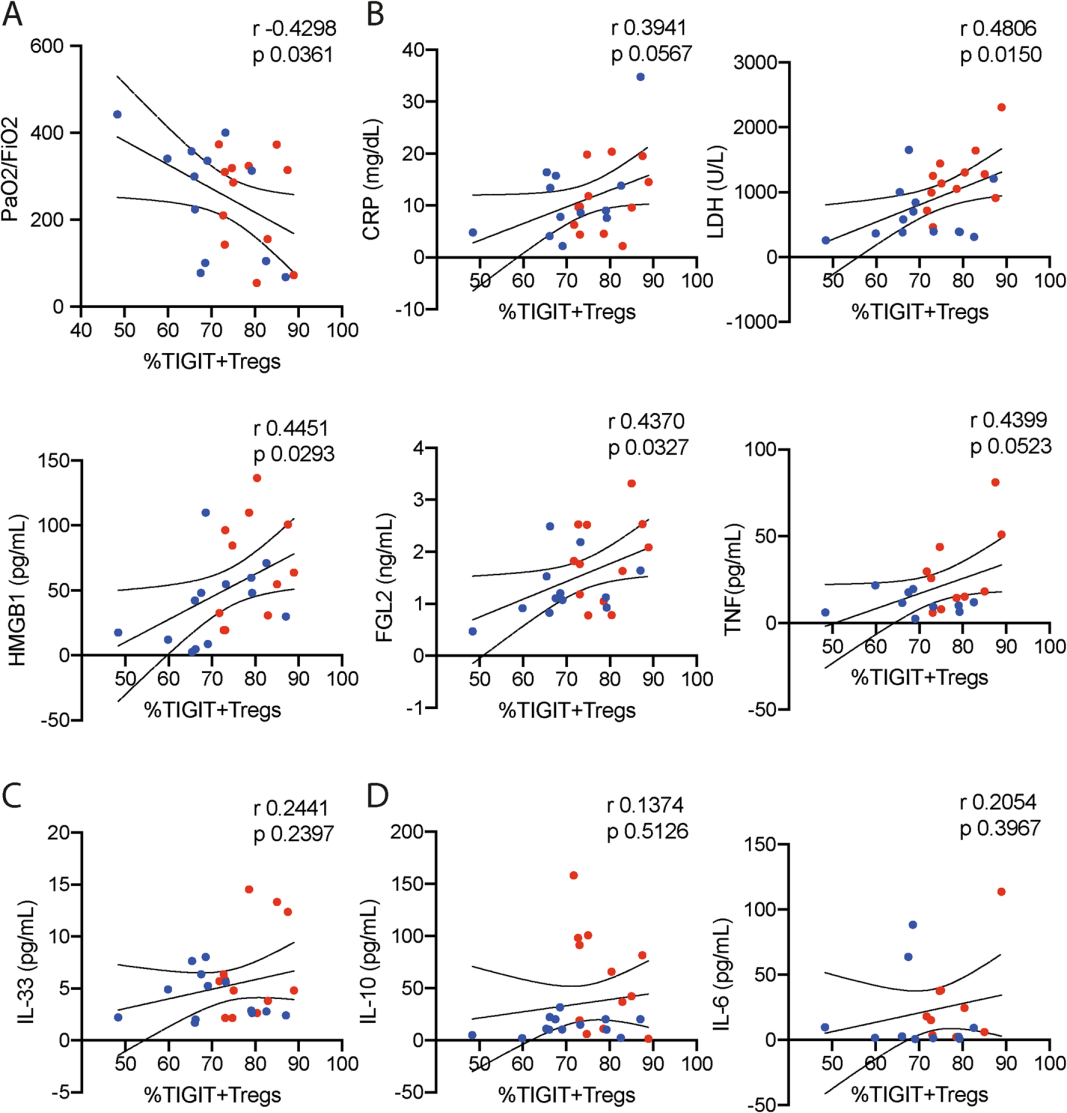
The prevalence of the TIGIT^+^Tregs correlates with lung dysfunction and increased inflammation. Correlation between the frequency of TIGIT^+^Treg subset among the FOXP3^+^Treg repertoire and the (**A**) PaO_2_/FiO_2_, and the plasmatic levels of (**B**) CRP, LDH, HMGB1, FGL2, and TNF; (**C**) IL-33; (**D**) IL-10 and IL-6. All parameters were analyzed during admission of 26 COVID-19 patients that did require mechanical ventilation during hospitalization. The blue-dots represent the survivors and red-dots is related to non-survivors. Spearman’s rank-order correlation (r) was calculated to describe correlations.

As our findings suggest that the TIGIT^+^FOXP3^+^T regulatory cells subset was more prevalent among patients with poor prognosis, the next round of experiments focused on evaluating some important cytokines in the plasma of patients that did require mechanical ventilation during admission, which were stratified in survivors and non-survivors for further correlation analysis. We have previously demonstrated that the alarmin IL-33, a cytokine released during tissue damage and necrosis^6^, has an important role in the TIGIT^+^FOXP3^+^ T regulatory cell expansion and Th2-biased immunity^5^. Although we did not observe any difference in the plasma levels of the Th2 cytokines IL-4 and IL-13 (Supplementary Fig. 2D), we could detect higher levels of IL-33 among the non-survivors (Table 1, Fig. 3A). The same was observed regarding the nuclear protein High Mobility Group Box 1 (HMGB1, Table 1, Fig. 3A), which is also released by necrotic and pyroptotic cells, and has an important role in intensifying inflammation and tissue damage^7,8^. The soluble form of the Fibrinogen-Like 2 (FGL2), which represents an important and distinct immunomodulatory mediator released by TIGIT^+^FOXP3^+^T regulatory cells^4^, and the IL-10 were also encountered in higher levels in the plasma of the non-survivor group (Fig. 3B). Even though we did not detect any difference in the IFN-γ and TNF levels comparing survivors with non-survivors (Fig. 3C), higher levels of IL-6 were detected among non-survivors (Fig. 3D). Although the PaO_2_/FiO_2_ per se was not able to predict the fatal outcome during hospital entrance (Table 1, Supplementary Fig. 2D), a negative correlation was detected between the PaO_2_/FiO_2_ and the frequency of TIGIT^+^FOXP3^+^T regulatory cell subset (Fig. 4A), indicating that the increased levels of these cells were associated with poor pulmonary functions. We also observed that the frequency of TIGIT^+^FOXP3^+^Treg subset had a significative positive correlation with the levels of LDH, HMGB1, and FGL2 (Fig. 4B) and showed a tendency to positively correlate with CRP and TNF, suggesting the prevalence of the TIGIT+FOXP3+Tregs in conditions with high inflammatory response and cell death. We did not observe any correlation between the frequencies of the TIGIT^+^FOXP3^+^T regulatory cells and the levels of D-Dimer and Ferritin (Supplementary Fig. 3A), as well as with the count of neutrophils, lymphocytes, monocytes, and platelets (Supplementary Fig. 3B). Despite the increased plasma levels of IL-33 among non-survivors during hospital entrance (Fig. 3A), we could not establish any correlation between the levels of IL-33 obtained from patients that develop respiratory dysfunction during hospitalization and their frequencies of the TIGIT+FOXP3+T regulatory cells (Fig. 4C), which suggest that another unknown mechanism could contribute to the prevalence of TIGIT^+^FOXP3^+^ Tregs in patients with poor prognosis. Similar results were observed regarding the levels of IL-10 and IL-6 (Fig. 4D). Collectively, the correlation analysis highlights the interaction between the highly inflammatory condition, cell death, and lung dysfunction in supporting the expansion of the TIGIT^+^FOXP3^+^Tregs subset.

### The intubated patients that did not survive presented a higher prevalence of bacteremia and nosocomial infections

**Figure 5:**
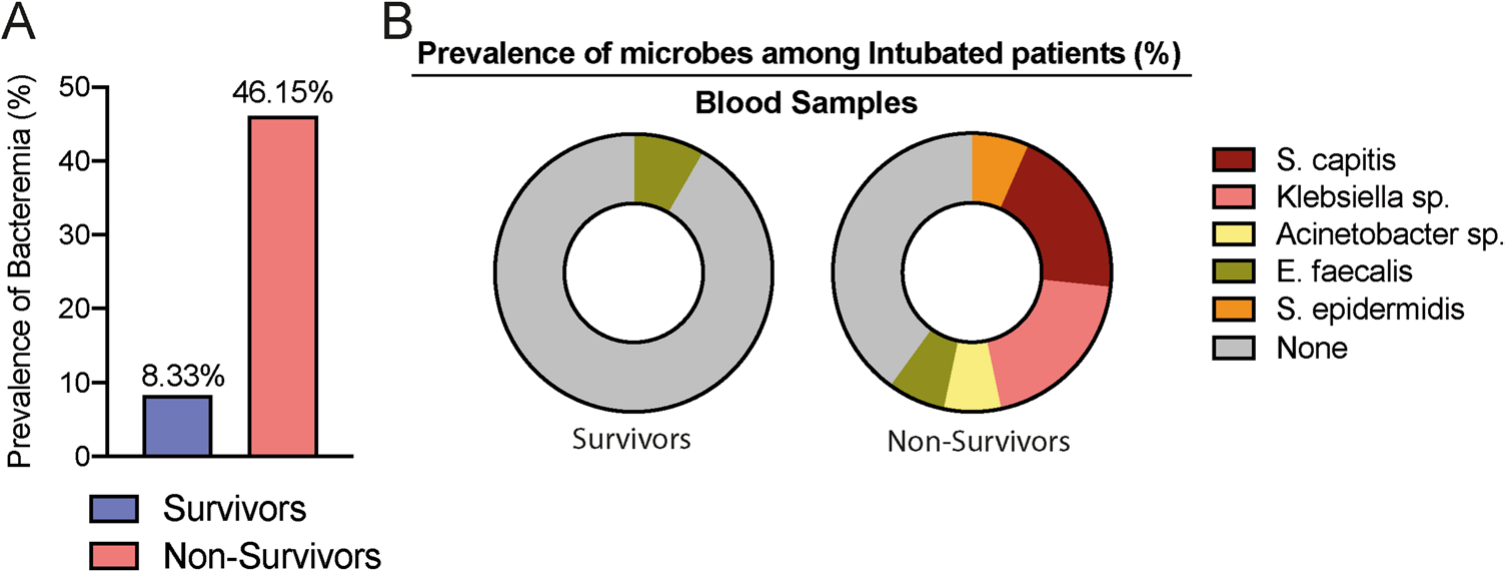
The COVID-19 fatal outcome was marked by increased bacteremia during hospitalization. The cohort of 26 COVID-19 patients that did require mechanical ventilation during hospitalization was evaluated in terms of (**A**) the prevalence of bacteremia among survivors (n = 13, blue bar) and non-survivors (n = 13, red bar). (**B**) Isolated microorganisms in blood culture among survivors (n = 13, left panel) and non-survivors (n = 13, right panel).

It has been demonstrated that the strong suppressive activity of the TIGIT^+^FOXP3^+^T regulatory cells subset on cellular immunity predisposes septic individuals to acquire opportunistic infections due to the development of immunosuppression^5^. Recognizing that COVID-19 shares some characteristics with sepsis^9,10^ and that ICU stay increases in 37% the risk of acquiring nosocomial and opportunistic infections^11^, we lastly investigated whether the intubated patients presented any secondary infection during hospitalization (Table 2). Corroborating our hypothesis, the prevalence of positive cultures for blood samples was 5.54 times higher among non-survivors (Fig. 5A).

**Table 2:** Culture results of patients on mechanical ventilation and the antibiotics used during hospitalization.

Patient	Group	Culture results (sites)	Antibiotics used
1	MV survivor	Candida parapsilosis (TSC), Candida tropicalis (UC), Proteus mirabilis (CTC), Serratia marcescens (CTC), Klebsiella pneumoniae (TSC)	Ceftriaxone, clarithromycin, meropenem, vancomycin, tigecycline, polymyxin B, fluconazole
2	MV survivor	Enterococcus faecalis (TSC), Trichosporon asahii (UC), Acinetobacter baumannii (TSC)	Ceftriaxone, clarithromycin, meropenem, vancomycin, fluconazole
3	MV survivor	Enterobacter cloacae (UC)	Ceftriaxone, clarithromycin, piperacillin-tazobactam
4	MV survivor	Klebsiella pneumoniae (CTC)	Ceftriaxone, azithromycin, piperacillin-tazobactam, meropenem, vancomycin
5	MV survivor	Candida albicans (UC)	Ceftriaxone, clarithromycin, piperacillin-tazobactam
6	MV survivor	Candida albicans (TSC)	Ceftriaxone, azithromycin, piperacillin-tazobactam
7	MV survivor	Enterococcus faecalis (BC), Candida tropicalis (UC), Candida albicans (TSC)	Ceftriaxone, clarithromycin, piperacillin-tazobactam, meropenem, vancomycin, polymyxin B, fluconazole
8	MV survivor	Serratia marcescens (TSC)	Ceftriaxone, clarithromycin
9	MV survivor	Candida tropicalis (TSC), Candida albicans (UC)	Ceftriaxone, azithromycin, piperacillin-tazobactam, vancomycin, fluconazole, micafungin
10	MV survivor	–	Ceftriaxone, clarithromycin
11	MV survivor	Klebsiella (UC)	Ceftriaxone, azithromycin, piperacillin-tazobactam
12	MV survivor	Staphylococcus aureus (TSC), Candida albicans (UC)	Ceftriaxone, clarithromycin, meropenem
13	MV survivor	–	Ceftriaxone, azithromycin
14	MV non survivor	–	Ceftriaxone, azithromycin
15	MV non survivor	Staphyloccocus epidermidis (BC), Staphylococcus capitis (BC), Klebsiella pneumoniae (BC)	Ceftriaxone, clarithromycin, meropenem, vancomycin, polymyxin B, amikacin, ceftazidime-avibactam
16	MV non survivor	Candida tropicalis (UC), Klebsiella pneumoniae (TSC), Acinetobacter baumannii (BAL)	Ceftriaxone, clarithromycin, meropenem, vancomycin, polymyxin B, fluconazole, gentamicin
17	MV non survivor	Acinetobacter baumannii (BC)	Ceftriaxone, clarithromycin
18	MV non survivor	Staphylococcus aureus (TSC)	Ceftriaxone, azithromycin, meropenem, vancomycin, polymyxin B, micafungin
19	MV non survivor	Acinetobacter baumannii (TSC), Serratia marcescens (TSC), Stenotrophomonas maltophilia (TSC), Staphylococcus epidermidis (CTC), Staphylococcus capitis (BC)	Ceftriaxone, azithromycin, meropenem, vancomycin, levofloxacin, gentamicin, polymyxin
20	MV non survivor	*Escherichia coli* (TSC) e Staphylococcus aureus (TSC)	Ceftriaxone, clarithromycin
21	MV non survivor	Staphylococcus capitis (BC), Klebsiella pneumoniae (BC), Enterococcus faecium (UC), Klebsiella pneumoniae (TSC), Candida glabrata (TSC), Pseudomonas aeruginosa (CTC), Staphylococcus epidermidis (CTC)	Ceftriaxone, clarithromycin, meropenem, vancomycin, ceftazidime-avibactam, voriconazole, polymyxin B, linezolid
22	MV non survivor	Syncephalastrum sp. (TSC)	Ceftriaxone, clarithromycin, oxacillin
23	MV non survivor	Enterococcus faecalis (BC), Pseudomonar aeruginosa (TSC)	Ceftriaxone, clarithromycin, cefepime, meropenem, ampicillin
24	MV non survivor	Candida tropicalis (UC)	Ceftriaxone, azithromycin, piperacillin-tazobactam, meropenem, vancomycin, amikacin, levofloxacin, fluconazole
25	MV non survivor	Klebsiella pneumoniae (BC and TSC), Staphylococcus epidermidis (CTC)	Ceftriaxone, clarithromycin, meropenem, vancomycin, polymyxin B
26	MV non survivor	Acinetobacter baumannii (TSC)	Ceftriaxone, clarithromycin

*MV* mechanical ventilation, *BC* blood culture, *UC* urine culture, *TSC* tracheal secretion culture, *CTC* catheter tip culture, *BAL* bronchoalveolar lavage culture.

As shown in Fig. 5B, we observed an accentuated diversity of pathogens in the blood culture from non-survivors. Quantitatively, a higher prevalence of *Staphylococcus capitis* (20%) and *Klebsiella pneumoniae* (20%), followed by *Staphylococcus epidermidis* (6.66%), *Enterococcus faecalis* (6.66%), and *Acinetobacter baumannii* (6.66%) was detected in blood samples from non-survivors; when only *Enterococcus faecalis* was detected in 8.33% of survivors. These data suggest that immune surveillance in terms of controlling localized infections was compromised among non-survivors, which presented a higher prevalence of deadly bacteremia.

## Discussion

FOXP3^+^ T regulatory cells' role in maintaining homeostasis by controlling autoimmunity and tissue repair is well recognized^12^. Their role in favoring the establishment of infections has also been demonstrated^13^. This concept can be observed in sepsis, where the strong inflammatory response is accompanied by compromised microbicidal immunity^14^. During sepsis, IL-33 released by damaged cells initiates a signaling cascade through its receptor ST2 on M2 macrophages that favors the expansion of the highly suppressive TIGIT^+^FOXP3^+^T regulatory cells^5^. Thus, the Th2-biased immunity initiated by IL-33 is involved with sepsis-induced immunosuppression^15^.

It has been observed that severe COVID-19 shares several features with sepsis, being also marked by cytokine storm, tissue damage, and signs of dysregulated immune response^2,9,10^. Herein, the refractory respiratory failure in patients with COVID-19 was associated with increased CRP, D-dimers, LDH, Ferritin, HMGB1, and IL-33, as well as higher numbers of circulating neutrophils, higher plasma levels of IL-6 and IL-10, and diminished numbers of lymphocytes and platelets. Furthermore, by assessing the IFN-γ, an essential mediator for antiviral immunity^16–21^, we also observed decreased levels of this cytokine in patients that required mechanical ventilation during hospitalization.

Confirming previous reports that hospitalized patients with COVID-19 present a reduction in both frequency and absolute numbers of FOXP3^+^Tregs compared to healthy individuals^2^, we also confirmed that the worst disease is associated with increased numbers of FOXP3^+^Tregs^13^. The expansion of the effector/memory phenotype of FOXP3^+^Tregs^13^ with a similar transcriptional profile of tumor-infiltrated Tregs^14^ was previously detected during COVID-19. Herein, the TIGIT^+^Tregs correlated with the magnitude of the inflammation and tissue damage, detected here by measuring the levels of LDH and HMGB1, as well as estimating the lung function by the PaO_2_/FiO_2_.

It has been extensively described that conditions of metabolic syndrome such as obesity, arterial hypertension, and diabetes are associated with worse prognosis in COVID-19^22^. In our study, the comorbidities mentioned above were also more frequent among patients with poor prognosis and fatal outcomes. Regarding influenza, another airway inflammatory disease caused by a viral infection, obese individuals are highly susceptible^23^, present an impairment of the production of IFN-γ by TCD8^+^ cells^24^, and compromised antiviral immunity^25^. Thus, it is plausible to hypothesize that the low-grade chronic inflammation observed in metabolic syndrome, also known as meta-inflammation, modulates the dynamics of the immune system in dealing with intracellular pathogens and viral infection. In mice, we have found that obesity, induced by 12 weeks of high-fat diet protocol, resulted in a 2-times expansion of the TIGIT^+^ Treg subset and did not interfere in the TIGIT^-^Treg compartment in secondary lymphoid organs (unpublished data). Therefore, we do not discard the involvement of the metabolic syndrome in favoring the TIGIT^+^Treg expansion during COVID-19. Further studies are required to elucidate the role of TIGIT^+^Tregs in obesity-associated infections.

Although the prominent increase of IL-33 and IL-10 among non-survivors, we could not detect any increase in the levels of the Th2-cytokines IL-4, IL-5, and IL-13 in their plasma at the moment of hospital admission. Considering the distinct function of TIGIT^+^Tregs in favoring Th2 immunity^4,5,26^, and that COVID-19 patients in ICU present signs of Th2-biased immunity^27,28^, we speculate that worse COVID-19 outcomes could be associated with impaired microbicidal immunity due to TIGIT^+^Tregs expansion.

Nosocomial or healthcare-associated infections are associated with higher morbidity, mortality, increased lengths of stay, and costs^29,30^. Such infections are frequently associated with immunosuppressed patients^31^, representing the second major cause of death due to COVID-19^10^. Cases of COVID-19-induced immunosuppression has been reported even in vaccinated individuals^9,32^. In our study cohort, the bacteremia was up to 6 times increased among non-survivors. Since *Staphylococcus capitis* and *Klebsiella pneumoniae* are commensal microorganisms found mainly in the skin and the gastrointestinal tract^33,34^, their prevalence in the blood of non-survivors suggests their immunocompromised condition due to COVID-19.

It is essential to mention that all parameters analyzed in this study were obtained before any therapeutic intervention, which eliminates the impact of anti-inflammatory drugs in our findings and justifies the low number of patients recruited in this study. Therapies aiming to modulate the effects of the cytokine storm, such as blocking the IL-6 receptor and abrogating the cytokine signaling pathway by using JAK inhibitors, have been associated with the recovery of clinically ill patients with COVID-19^35–37^. Thus, we speculate that controlling the extension of tissue damage and inflammation could be beneficial in attenuating the TIGIT^+^Treg expansion and consequently avoiding deadly nosocomial infections during ICU stay. Further studies are required to verify whether another ICU-related disease than sepsis and COVID-19 could improve the TIGIT^+^Treg expansion and test its association with nosocomial infections.

The limitations of the present study concern the need for more data involving the measurement of the TIGIT^+^Tregs during ICU stay and the follow-up of the patients to determine the burden of the secondary infection and its correlation with TIGIT. However, supported by previous reports that TIGIT blockade can restore the effector functions of TCD8^+^ cells during experimental sepsis^38^ and experimental model of chronic viral infection by LCMV^39^, we suggest that therapies aiming the TIGIT blockade would be beneficial in preventing nosocomial and opportunistic infections in hospitalized COVID-19 patients.

## Conclusion

The high levels of the TIGIT^+^Tregs during hospitalization due to COVID-19 are accompanied by intense inflammatory response, inflammatory cell death, lung dysfunction, and hospital-acquired bacteremia.

### Supplementary Information

Supplementary Figure 1.

Supplementary Figure 2.

Supplementary Figure 3.

Supplementary Information 4.

## Data Availability

The datasets used and/or analyzed during the current study are available from the corresponding author upon reasonable request.

## References

[1] Zhou F (2020). Clinical course and risk factors for mortality of adult inpatients with COVID-19 in Wuhan, China: a retrospective cohort study. Lancet.

[2] Yang X (2020). Clinical course and outcomes of critically ill patients with SARS-CoV-2 pneumonia in Wuhan, China: a single-centered, retrospective, observational study. Lancet Respir. Med..

[3] WHO Coronavirus (COVID-19) Dashboard at https://covid19.who.int.

[4] Fathi M, Markazi-Moghaddam N, Ramezankhani A (2019). A systematic review on risk factors associated with sepsis in patients admitted to intensive care units. Aust. Crit. Care..

[5] de Guo W (2020). Diabetes is a risk factor for the progression and prognosis of COVID-19. Diabetes Metab. Res. Rev..

[6] Gao F (2020). Obesity is a risk factor for greater COVID-19 severity. Diabetes Care.

[7] Sattar N, McInnes IB, McMurray JJV (2020). Obesity is a risk factor for severe COVID-19 infection. Circulation.

[8] Matsushita K (2020). The relationship of covid-19 severity with cardiovascular disease and its traditional risk factors: a systematic review and meta-analysis. Global Heart.

[9] Baskaran V (2021). Co-infection in critically ill patients with COVID-19: an observational cohort study from England. J. Med. Microbiol..

[10] Bardi T (2021). Nosocomial infections associated to COVID-19 in the intensive care unit: clinical characteristics and outcome. Eur. J. Clin. Microbiol. Infect. Dis..

[11] Sakaguchi S (2006). Foxp3+CD25+CD4+ natural regulatory T cells in dominant self-tolerance and autoimmune disease. Immunol. Rev..

[12] Chen G (2020). Clinical and immunological features of severe and moderate coronavirus disease 2019. J. Clin. Invest..

[13] Rahimzadeh M, Naderi N (2021). Toward an understanding of regulatory T cells in COVID-19: A systematic review. J. Med. Virol..

[14] Galván-Peña S (2021). Profound treg perturbations correlate with COVID-19 severity. Proc. Natl. Acad. Sci..

[15] Joller N (2014). Treg cells expressing the coinhibitory molecule TIGIT selectively inhibit proinflammatory Th1 and Th17 cell responses. Immunity.

[16] Dixon KO (2018). Functional anti-TIGIT antibodies regulate development of autoimmunity and antitumor immunity. J. Immunol..

[17] de Lima MHF (2022). Sepsis-induced immunosuppression is marked by an expansion of a highly suppressive repertoire of FOXP3+ T-regulatory cells expressing TIGIT. J. Infect. Dis..

[18] Parra B, Hinton DR, Marten NW (1999). IFN-γ is required for viral clearance from central nervous system oligodendroglia. J. Immunol..

[19] Orange JS, Biron CA (1996). An absolute and restricted requirement for IL-12 in natural killer cell IFN-gamma production and antiviral defense. Studies of natural killer and T cell responses in contrasting viral infections. J. Immunol..

[20] Whitmire JK, Tan JT, Whitton JL (2005). Interferon-γ acts directly on CD8+ T cells to increase their abundance during virus infection. J. Exp. Med..

[21] Rhein BA, Powers LS, Rogers K (2015). Interferon-γ inhibits ebola virus infection. PLoS Pathog..

[22] Andrade FB, Gualberto A, Rezende C, Percegoni N, Gameiro J, Hottz ED (2021). The weight of obesity in immunity from influenza to COVID-19. Front. Cell. Infect. Microbiol..

[23] Karki S, Muscatello DJ, Banks E, MacIntyre CR, McIntyre P, Liu B (2018). Association between body mass index and laboratory-confirmed influenza in middle aged and older adults: A prospective cohort study. Int. J. Obes..

[24] Karlsson EA, Sheridan PA, Beck MA (2010). Diet-induced obesity impairs the T cell memory response to influenza virus infection. J. Immunol..

[25] Smith AG, Sheridan PA, Harp JB, Beck MA (2007). Diet-induced obese mice have increased mortality and altered immune responses when infected with influenza virus12. J. Nutr..

[26] Lozano E, Joller N, Cao Y, Kuchroo KV, Hafler DA (2013). The CD226/CD155 interaction regulates the proinflammatory (Th1/Th17)/anti-inflammatory (Th2) balance in humans. J. Immunol..

[27] Rodriguez, L., Pekkarinen, P. T. & Lakshmikanth, T., *et al*. Systems-level immunomonitoring from acute to recovery phase of severe COVID-19. CR Med. 1(5) (2020). https://www.cell.com/cell-reports-medicine/abstract/S2666-3791(20)30099-9. 10.1016/j.xcrm.2020.100078 PMC7405891 32838342

[28] Roncati L, Nasillo V, Lusenti B, Riva G (2020). Signals of Th2 immune response from COVID-19 patients requiring intensive care. Ann. Hematol..

[29] Benenson S, Cohen MJ, Schwartz C, Revva M, Moses AE, Levin PD (2020). Is it financially beneficial for hospitals to prevent nosocomial infections?. BMC Health Serv. Res..

[30] Prowle JR, Echeverri JE, Ligabo EV (2011). Acquired bloodstream infection in the intensive care unit: Incidence and attributable mortality. Crit. Care.

[31] García-Rodríguez JF, Mariño-Callejo A (2023). The factors associated with the trend in incidence of Bacteraemia and associated mortality over 30 years. BMC Infect. Dis..

[32] Seffah, K., Agyeman, W. Y., Seffah, K. D. & Agyeman, W. Y. A suspected case of COVID-19-induced immunosuppression. Cureus 14(12) (2022). https://www.cureus.com/articles/127034-a-suspected-case-of-covid-19-induced-immunosuppression. 10.7759/cureus.32227 PMC9812533 36620840

[33] Kang J, Tackling G, Patel D, Akella J (2020). Double bacteremia by rare pathogens: Staphylococcus capitis and leuconostoc species. Chest.

[34] Papadimitriou-Olivgeris M, Bartzavali C, Georgakopoulou A (2021). Mortality of pandrug-resistant klebsiella pneumoniae bloodstream infections in critically Ill patients: A retrospective cohort of 115 episodes. Antibiotics.

[35] Rokni M, Hamblin MR, Rezaei N (2020). Cytokines and COVID-19: Friends or foes?. Hum. Vaccines Immunother..

[36] Interleukin-6 receptor antagonists in critically Ill patients with Covid-19. N. Engl. J. Med. **384**(16), 1491–1502 (2021). 10.1056/NEJMoa2100433. 10.1056/NEJMoa2100433 PMC7953461 33631065

[37] Baricitinib plus Remdesivir for Hospitalized Adults with Covid-19 | NEJM. 10.1056/NEJMoa2031994. 10.1056/NEJMoa2031994 PMC7745180 33306283

[38] Sun Y, Ding R, Chang Y, Li J, Ma X (2021). Immune checkpoint molecule TIGIT manipulates T cell dysfunction in septic patients. Int. Immunopharmacol..

[39] Schorer M, Rakebrandt N, Lambert K (2020). TIGIT limits immune pathology during viral infections. Nat. Commun..

