# Supplementary figures and images for: The TIGIT+ T regulatory cells subset associates with nosocomial infection and fatal outcome in COVID-19 patients under mechanical ventilation
Source: Sci Rep. 2023 Aug 21;13:13599. doi: 10.1038/s41598-023-39924-7 (PMC10442317; doi:10.1038/s41598-023-39924-7)

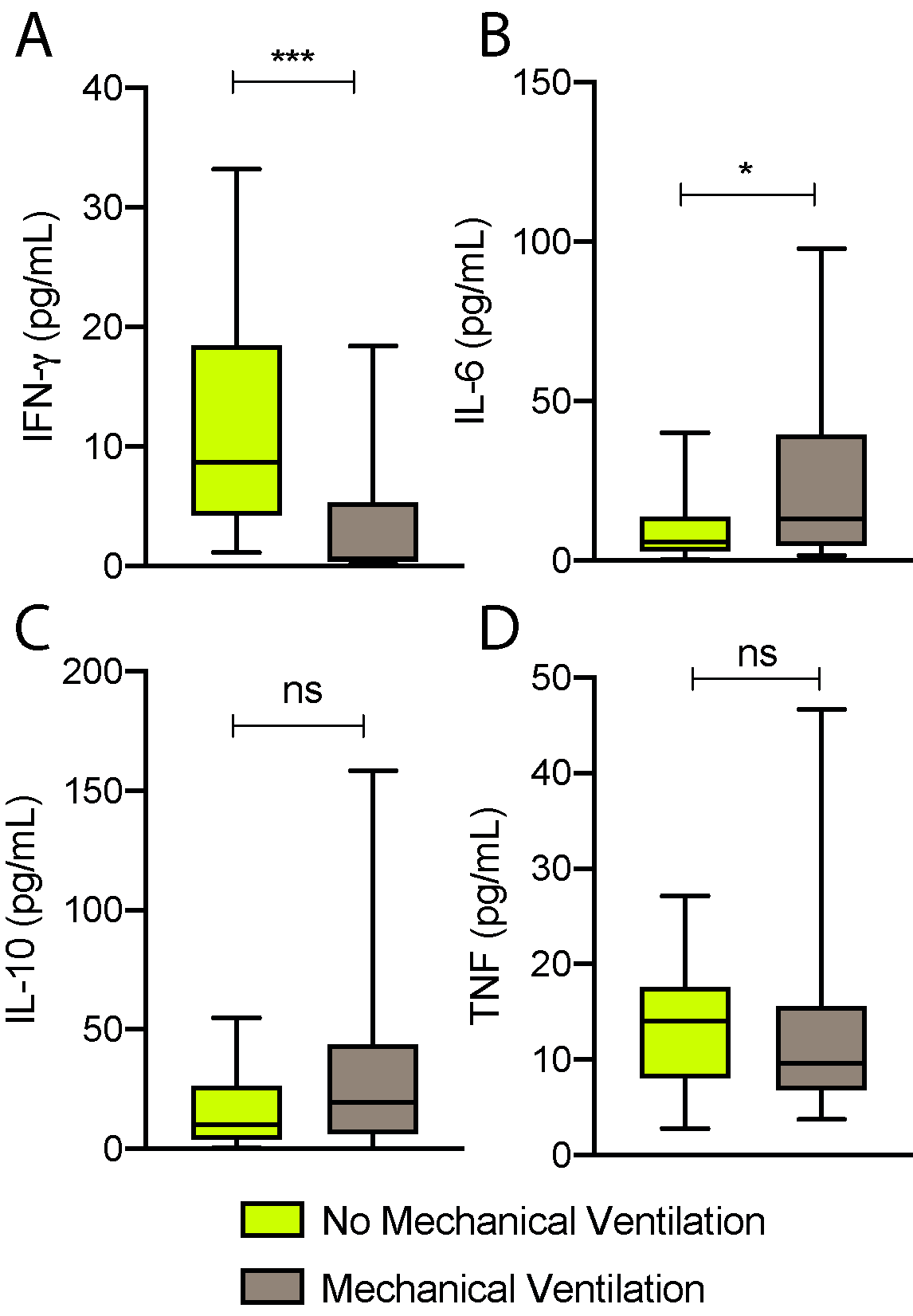

Supplement: Supplementary file 1 — Supplementary Figure 1. [file 41598_2023_39924_MOESM1_ESM.tif]

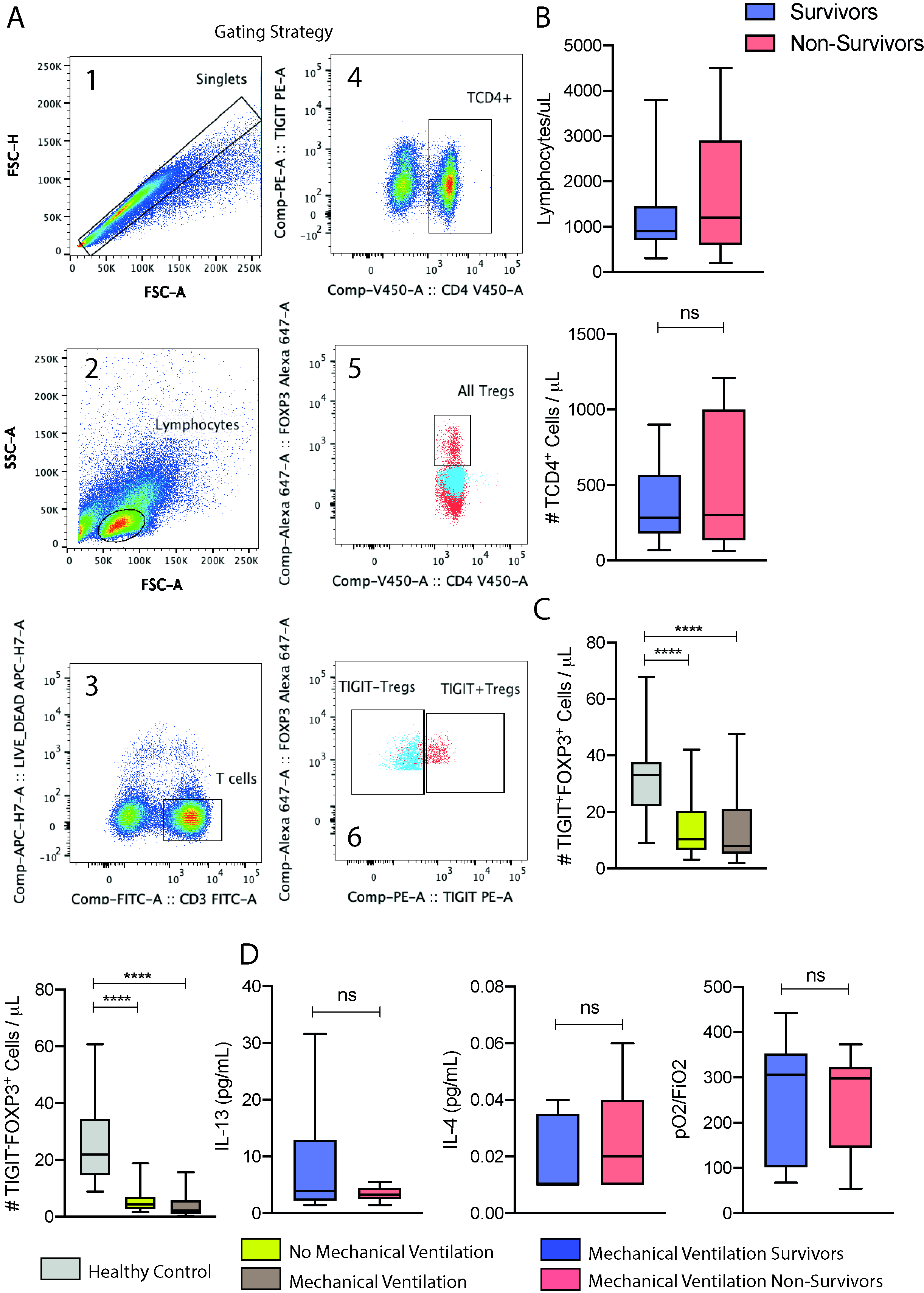

Supplement: Supplementary file 2 — Supplementary Figure 2. [file 41598_2023_39924_MOESM2_ESM.tif]

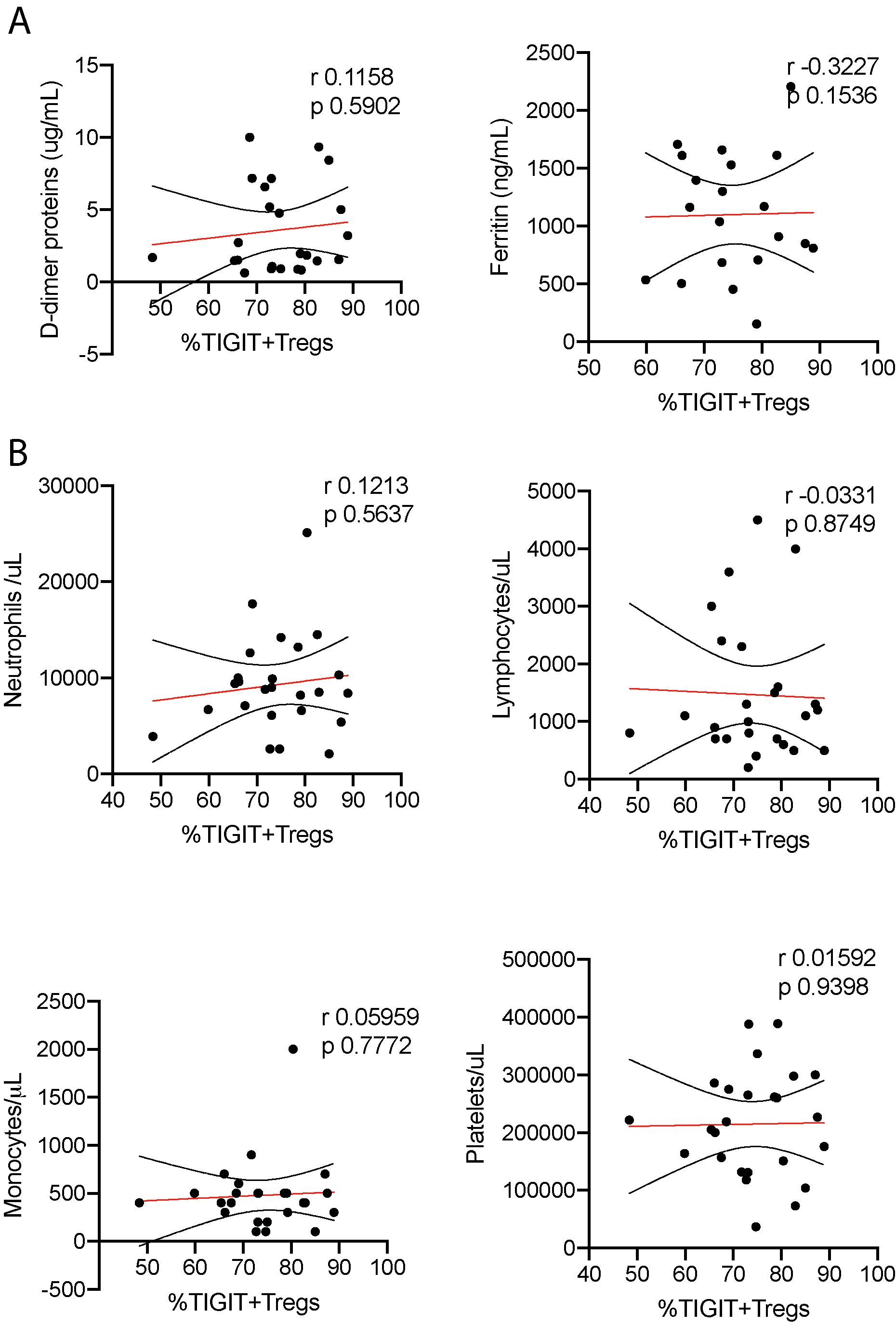

Supplement: Supplementary file 3 — Supplementary Figure 3. [file 41598_2023_39924_MOESM3_ESM.tif]
