# Supplementary material for: The TIGIT+ T regulatory cells subset associates with nosocomial infection and fatal outcome in COVID-19 patients under mechanical ventilation
Source: Sci Rep. 2023 Aug 21;13:13599. doi: 10.1038/s41598-023-39924-7 (PMC10442317; doi:10.1038/s41598-023-39924-7)
Supplement: Supplementary file 4 — Supplementary Information 4. [file 41598_2023_39924_MOESM4_ESM.docx]

**Supplementary Figure 1: The mechanical ventilation requirement was associated with diminished circulating levels of IFN-γ and improved levels of IL-6.** Blood samples of 72 COVID-19 patients collected at admission were evaluated according to their cytokines levels in the plasma applying the Luminex methodology. The data was plotted according to the need for mechanical ventilation during hospitalization and expressed in picograms per milliliter (pg/mL). Patients that did not require mechanical ventilation are represented by the yellow bars (n=46), and the intubated patients are represented by the brown bars (n=26). **(A)** Interferon-gamma, IFN-γ; **(B)** Interleukin-6, IL-6; **(C)** Interleukin-10, IL-10; **(D)** Tumor necrosis factor, TNF. Statistical significance was determined by either the unpaired Student t test for data that reached normal distribution, and the Mann–Whitney test for not normally distributed data. *p<0.05

**Supplementary Figure 2: Flow cytometry gating parameters applied for the immunophenotyping of the FOXP3+Tregs repertoire and the TIGIT+Tregs subset. (A)** The gating strategy parameters were made applying the following order of analysis: 1- Singlets; 2- Lymphocytes; 3- Live T cells; 4- TCD4+cell; 5- FOXP3+Treg repertoire; 6- TIGIT+ and TIGIT- Treg subsets. The Fluorescence minus one (FMO) control were made to establish the positive and negative staining for FOXP3 and TIGIT, and are represented as the blue dots in 5 and 6. **(B)** The absolute number of lymphocytes, TCD4+ cells, **(C)** TIGIT+FOXP3+Tregs, and TIGIT-FOXP3+Tregs were measured in the blood. The plasma levels of **(D)** Interleukin-13, IL-13; and Interleukin-4, IL-4; PaO2/FiO2 measured during admission. Healthy Controls are represented by gray bars (n=18), patients that did not require mechanical ventilation are represented by the yellow bars (n=18), and the intubated patients are represented by the brown bars (n=26). The intubated patients were stratified in survivors (blue bars, n=13) and non-survivors (red bars, n=13). Statistical significance was determined by either the one-way ANOVA followed by Tukey’s post hoc test, or the unpaired Student t test for data that reached normal distribution, and the Mann–Whitney test for not normally distributed data. *p<0.05

**Supplementary Figure 3: Correlation between the prevalence of the TIGIT+Tregs and laboratory parameters of COVID-19 patients that required mechanical ventilation during hospitalization.** Correlation between the frequency of TIGIT+Treg subset among the FOXP3+Treg repertoire and the **(A)** D-dimer proteins and Ferritin; **(B)** Neutrophils, Lymphocytes, Monocytes, and Platelets. All parameters were analyzed during admission of 26 COVID-19. Spearman’s rank-order correlation (r) was calculated to describe correlations.
